# Exploiting dispersion of higher-order-modes using M-type fiber for application in mid-infrared supercontinuum generation

**DOI:** 10.1038/s41598-019-44951-4

**Published:** 2019-06-12

**Authors:** D. Jain, C. Markos, T. M. Benson, A. B. Seddon, O. Bang

**Affiliations:** 1 DTU Fotonik, Department of Photonics Engineering, Technical University of Denmark, Lyngby, Denmark; 2 George Green Institute for Electromagnetics Research, University of Nottingham, Nottingham, NG7 2RD UK; 3 NKT Photonics A/S, Blokken 84, 3460 Birkerød, Denmark; 4 NORBLIS IVS, Virumgade 35D, 2830 Virum, Denmark; 5 School of Physics and The University of Sydney Nano Institute, The University of Sydney, Sydney, NSW 2006 Australia

**Keywords:** Fibre optics and optical communications, Fibre lasers

## Abstract

M-type fibers have the exceptional property that the higher-order LP_0n_ modes are core-confined and easily excited, while the LP_01_ and other modes are confined to a high-index ring surrounding the core, so they are not easily excited. This has profound consequences for mid-infrared supercontinuum sources, where the high zero-dispersion wavelength of chalcogenide and ZBLAN fibers means that exotic pump sources have so far been necessary. We show here that in chalcogenide and ZBLAN M-type fibers the lower ZDW of the core-confined higher-order LP_02_ mode can be in the range of 2 to 3 µm (around 1.55 µm), while the fiber still has a large core diameter and thus supports high average power. This will allow established pump laser technology to be used in future high-power mid-infrared supercontinuum sources.

## Introduction

**Figure 1:**
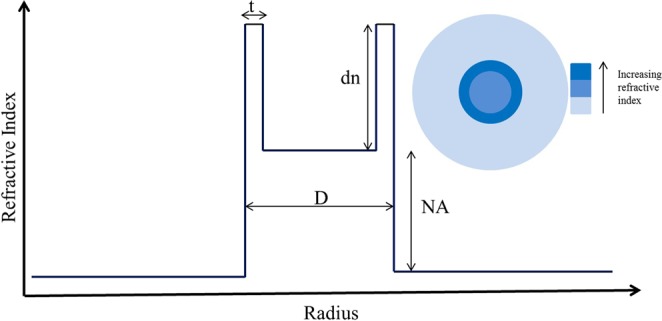
Refractive index profile of the M-type fiber with characteristic parameters t, dn, D, and NA. The inset shows the end facet of an M-type fiber.

Optical fibers have revolutionized human life via their utility in several applications, such as communication, sensing, light sources, and medical equipment etc. Most of the properties of an optical fiber depend on its refractive index profile (RIP). There are different types of optical fibers categorized according to their RIP, such as the standard step-index fiber (SIF), graded-index fiber (GIF), and photonic crystal fiber (PCF). The M-type optical fiber is another fiber with a distinct RIP, which is perhaps not so well-known, even though it has exceptional properties^1,2^. Figure 1 presents the RIP of an M-type fiber, which has an additional high index ring with thickness t surrounding the core of a conventional SIF. Due to the shape of the RIP, resembling the letter “M”, the fiber has become known as the M-type fiber, similar to a W-type fiber^1^.

The M-type fiber has special properties, which can be useful for several applications, where standard fibers have limited use. M-type fibers with rather broad rings (large t) and shallow refractive index dips (small dn) have been exploited in the past for high-power laser applications thanks to their flat fundamental mode profile^3–5^. This flat (top-hat) profile leads to a dramatic increase in effective mode area^3–5^, which is useful in avoiding non-linear effects. More recently, M-type fibers with narrow rings (small t) and larger refractive index dips (large dn) were shown to be suitable for exciting higher-order modes (HOMs) such as the LP_02_, as core-confined modes, while confining the LP_01_ and other modes to the high index ring, so that they are not easily excited^6–11^. Using an extra refractive index structure in the form of a depressed cladding region it was shown that using the core-confined LP_02_ mode the zero-dispersion wavelength (ZDW) could be pushed below 1 µm in a silica fiber^8^. It was further stated that had the core index and the index of the ring been significantly higher than that of silica, then the extra depressed cladding structure would not have been necessary^8^.

The main scope of this paper is to explore the M-type fiber design for shifting the ZDW of non-silica fibers, like chalcogenide and ZBLAN fibers, towards shorter wavelengths for application in mid-infrared (IR) supercontinuum (SC) generation. It is pertinent to note that mid-IR wavelength is extremely useful wavelength region as several chemical compounds and molecules display unique and strong mid-IR spectral finger prints in this region. Recently, mid-IR fiberized SC sources have shown their utility in different application such as Optical Coherence Tomography^12^, Tissue Imaging^13^, and as an alternative option of Synchrotrons^14^.

It is most efficient to pump the fiber near the ZDW for SC generation^15,16^. However, the ZDW of a material is fixed, so, in order to match it with available pump sources, a strong additional contribution from waveguide dispersion is generally required. Today’s SC sources use a silica PCF to provide the required ZDW and tight confinement, but silica PCFs are difficult to fabricate as they involve a complex stack and draw process and fiber handling (such as cleaving and splicing) is difficult due to the presence of air-holes^17^. Furthermore, it is even more challenging to fabricate PCFs based on chalcogenide^18,19^ and in particular ZBLAN glasses^20,21^ that are required for mid-IR SC sources^22^.

Even if the PCF technology can be matured the chalcogenide and ZBLAN glasses have such a long material ZDW that moving the ZDW sufficiently down to for example 1.5 μm to use Erbium pump lasers required so small cores that power handling becomes a critical issue^23^. Chalcogenide SIFs can be made with a very high NA above 1, but the dispersion of their fundamental LP_01_ mode is nevertheless not appropriate for broadband SC generation^24^.

An answer to the problems could be to use the M-type fiber and the fact that HOMs in conventional SIFs, such as the LP_02_ and LP_03_ modes, have a shorter ZDW than the LP_01_ mode^25,26^. As stated above it is possible to excite the LP_02_ or LP_03_ mode in an M-type fiber, while confining major fraction of power of the lower order modes (LOMs) such as LP_01_, LP_11_, and LP_21_ to the ring only. In this paper, we therefore explore the properties of M-type fibers providing a short ZDW in large-core SIFs for optimum SC generation. To the best of our knowledge, this is the first report showing the potentially very advantageous use of an M-type fiber in high-power SC generation in general and mid-IR SC generation in particular.

## Working Principle of the M-type Fiber

Figure 1 shows the RIP of the M-type fiber and the notations used in this paper: D is the core diameter, t is the thickness of the high-index ring, NA is the numerical aperture (NA) of the core with respect to the cladding, and dn is the refractive index difference between the high index ring and the core.

The working principle of an M-type fiber is different from a conventional SIF due to the different RIP. The special M-type RIP brings two features to an M-type fiber. The first one is how the spatial distribution of the modes varies with wavelength. The second one is the hybrid guiding mechanism consisting of both total internal reflection and anti-resonance guidance (similar to bandgap guidance). In an M-type fiber, the spatial distribution of the mode varies dramatically with wavelength, hence it is possible to confine the major power fraction of LP_0n_ modes to the core, while the major power power of LP_01_ mode and other modes are confined to the ring. Numerical simulations on these fibers have been performed with the full-vectorial finite element method (FEM) based software “COMSOL”. However, scalar LP notations have been used to follow conventional notations as the index difference between core and cladding is not high enough to cause any considerable difference. For dispersion calculations of LP_01_ and LP_02_ modes, a perfect degeneracy for both orthogonal polarizations has been assumed.

**Figure 2:**
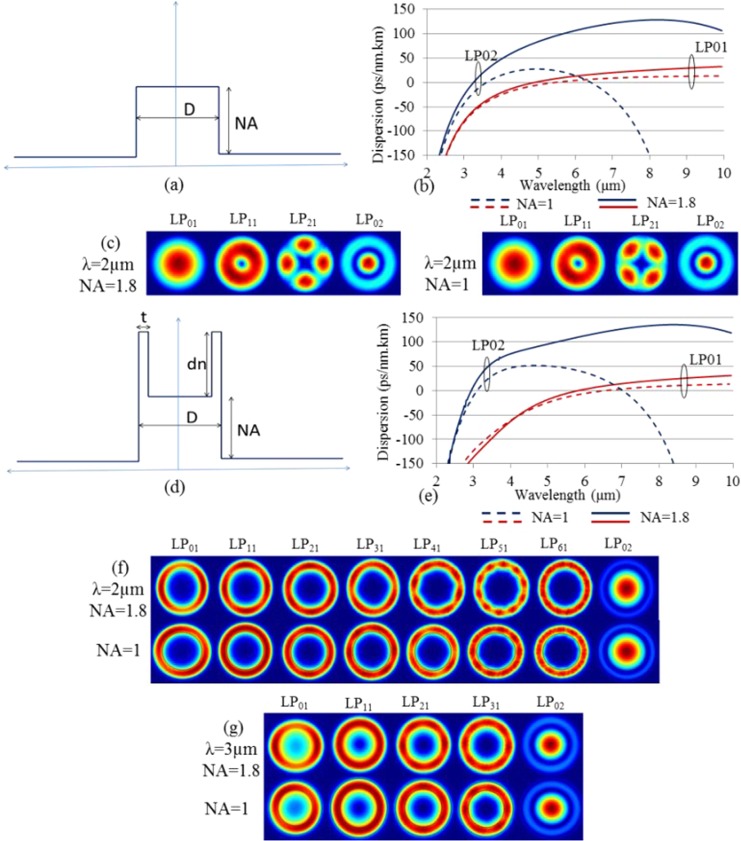
(**a**) Refractive index profile of a SIF, (**b**) calculated dispersion of the LP_01_ and LP_02_ modes of two chalcogenide SIFs with D = 12 µm and NA = 1 and 1.8, respectively, (**c**) electric field intensity profile at λ = 2 µm of guided modes in the SIFs, (**d**) RIP profile of an M-type fiber, (**e**) calculated dispersion of the LP_01_ and LP_02_ modes of two chalcogenide M-type fibers with D = 12 µm, dn = 0.05, t = 2 µm, and NA = 1 and 1.8, respectively, (**f**) electric field intensity profile of guided modes at λ = 2 µm, and (**g**) electric field intensity profile of guided modes at 3 µm for both M-type fibers.

Figure 2(a) shows the RIP of a SIF and Fig. 2(b) shows the calculated total dispersion of the LP_01_ and LP_02_ modes, for two different fibers with core diameter D = 12 µm and NA = 1 and 1.8. Here we used two glasses composed of As_40_Se_60_ (n_1_) and Ge_10_As_23.4_Se_66.6_ (n_2_), respectively, whose refractive indices were obtained from the refractive index measurement reported in^27^. The Sellmeier equations used in our studies are:

1 $${n}_{1}^{2}=1+\frac{6.558{\lambda }^{2}}{{\lambda }^{2}-0.09456}+\frac{0.8558{\lambda }^{2}}{{\lambda }^{2}-1669}+\frac{0.08432{\lambda }^{2}}{{\lambda }^{2}-0.5489}$$

2 $${n}_{2}^{2}=1+\frac{0.2325{\lambda }^{2}}{{\lambda }^{2}+0.3793}+\frac{0.8622{\lambda }^{2}}{{\lambda }^{2}-1709}+\frac{5.435{\lambda }^{2}}{{\lambda }^{2}-0.1036}$$

We consider SIFs composed of a core material with n_core_ = n_1_ and a cladding material with n_clad_ = n_2_, which at 3 µm has an NA of ~0.99, calculated from the standard relation:-

3 $${{\rm{NA}}}^{2}(\lambda )={n}_{1}^{2}(\lambda )-{n}_{2}^{2}(\lambda )$$

This is termed the NA=1 SIF and is the fiber used in^28^. However, we need several different NAs to observe the impact of NA on the ZDW. In order to increase the NA, we decreased the refractive index of the cladding by a factor independent of wavelength:-

4 $${{\rm{NA}}}^{2}(\lambda )={n}_{1}^{2}(\lambda )-{({n}_{2}(\lambda )-X)}^{2}$$

Figure 2(c) shows the electric field intensity profile of various modes of NA = 1 and 1.8 SIFs. In order to extend this study to an M-type fiber, we modified the SIF to an M-type fiber with the same basic parameters by increasing the index by dn = 0.05 in a small outer ring of thickness t = 2 µm of the core of the SIF (c.f. Fig. 2(d)). The core diameter and NA is the same for the two fiber types (D = 12 µm and NA = 1 or 1.8) and the new additional parameters are fixed at t = 2 µm and dn = 0.05. We obtained the refractive index of the high index ring as n_ring_(λ) = n_1_(λ) + dn. Figure 2(e) shows the calculated dispersion of the M-type fibers for the LP_01_ and LP_02_ modes within the same wavelength range as before. Figure 2(f,g) show the electric field intensity profile of several guided modes at 2 and 3 µm wavelengths for both fibers, respectively.

It is evident that the first mode residing in the central core region is the LP_02_ mode for both M-type fibers with NA = 1.8 and 1. Hence, it is possible to excite the LP_02_ mode while avoiding the modes residing primarily in the ring. In this way, one can exploit the ZDW of the LP_02_ mode (3.14 µm and 2.94 µm for NA = 1 and NA = 1.8, respectively), which is much shorter than the ZDW of the LP_01_ mode (6.59 µm and 5.81 µm for NA = 1 and 1.8, respectively) as shown in Fig. 2(e). It is important to note that, for a SIF with the same core diameter D = 12 µm with NA = 1 and NA = 1.8, the ZDW of the LP_01_ mode is also much higher at 5.48 µm and 4.9 µm, respectively, as shown in Fig. 2(b).

**Figure 3:**
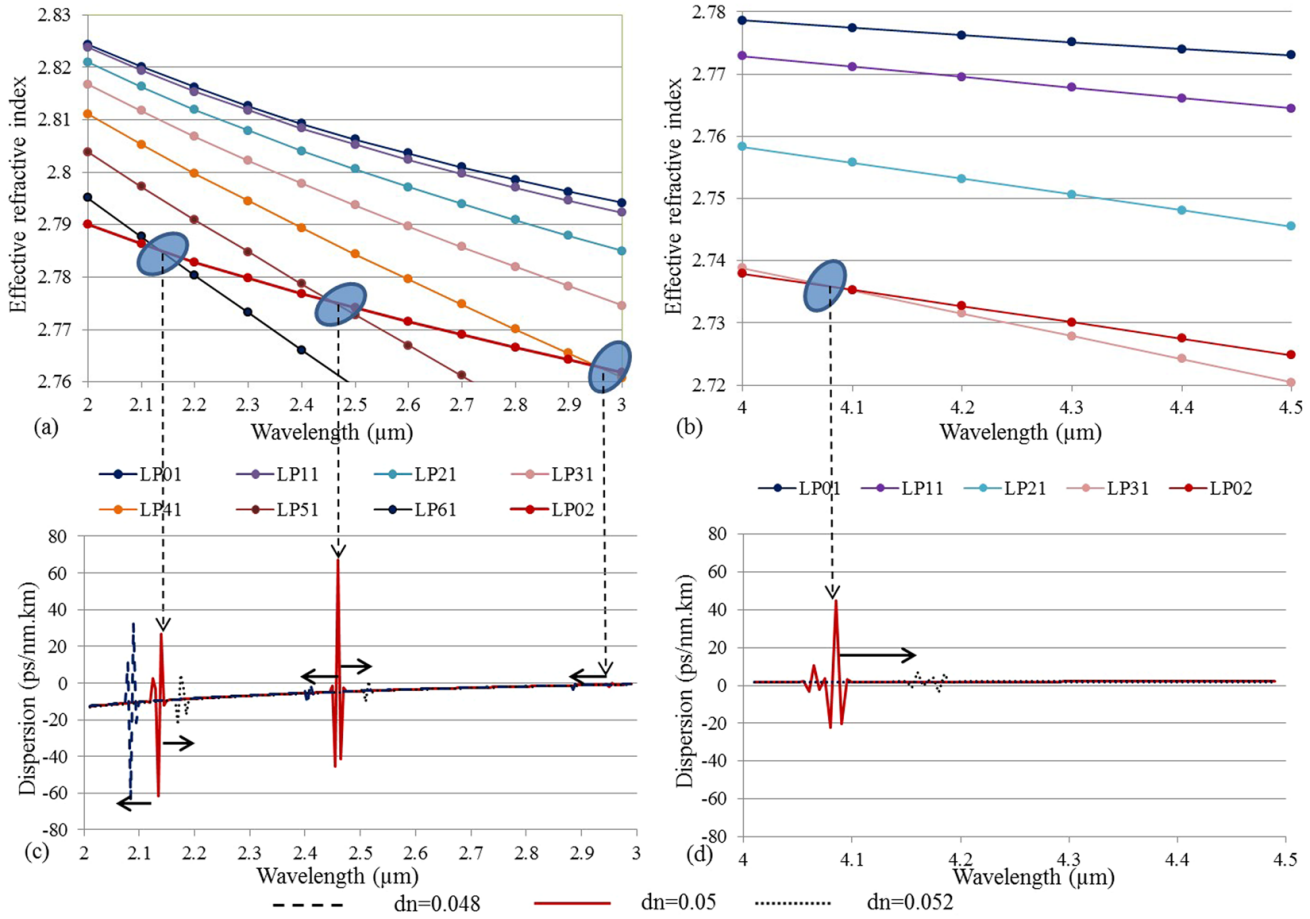
Variation of the effective refractive indices with respect to wavelength of several modes of the chalcogenide M-type fiber. (**a**) shows resonant coupling between the LP_02_ mode and the LP_61_, LP_51_, and LP_41_ modes, (**b**) shows resonant coupling between LP_02_ and LP_31_. The core diameter (D) is 12 µm, ring thickness (t) is 2 µm, NA is 1, and dn is 0.05. (**c**) and (**d**) shows the calculated dispersion of the LP_02_ mode for different dn. Still D = 12 µm, t = 2 µm, and NA = 1.

Figure 3(a,b) shows the variation of the effective refractive index of different modes of an M-type fiber (D = 12 µm, NA = 1, t = 2 µm, and dn = 0.05). With increasing wavelength, the effective refractive indices of the LP_61_, LP_51_, LP_41_, and LP_31_ modes rapidly decrease and cross the LP_02_ mode leading to resonant coupling at the specific wavelengths. This leads to local fluctuations in the dispersion profile of the LP_02_ mode as shown in Fig. 3(c,d), which may have implications for the SC generation. The similar resonant couplings effects were observed in silica photnic bandgap fiber^6^ and silica M-type fiber^11^. In fact, in silica M-type fiber the effect was exploited for pulse compression^11^. It is therefore important that one can shift these crossings by varying the fiber parameters. If one for example changes dn of the ring while keeping all other parameters to be the same then this changes the effective refractive indices of the modes in the ring and shifts the resonant coupling wavelength between these ring-confined modes and the LP_02_ mode, as seen in Fig. 3(c,d). Decreasing (increasing) dn by 0.002 for example shifts the 2.14 μm resonance by 0.05 μm (0.035 μm). Studying how these narrow-band crossings influence the SC generation will be the subject of future investigations, here we limit ourselves to a thorough study of the mode properties and dispersion.

**Figure 4:**
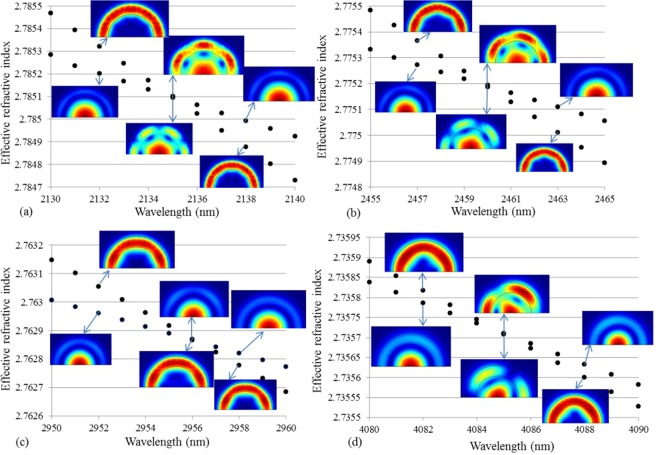
Variation of the effective refractive indices and profile of the electric field intensity for different couplings encountered by the LP_02_ mode in the chalcogenide M-type fiber. (**a**) LP_61_ and LP_02_, (**b**) LP_51_ and LP_02_ (**c**) LP41 and LP_02_, and (**d**) LP_31_ and LP_02_. The core diameter (D) is 12 µm, ring thickness (t) is 2 µm, NA is 1, and dn is 0.05.

Figure 4 explores these couplings in detail by showing the profile of the electric field intensity of the modes at different wavelengths close to the crossing. Away from the crossing the modes are all radially symmetric and therefore not orthogonal, which means that they couple strongly close to the crossing. Resonant coupling is thus clearly observed, which confirms a guiding mechanism similar to that found in photonic bandgap fibers (PBGFs), although total internal reflection guidance dominates^8,29^. It is pertinent to note that because there is only one resonator (the high-index ring) the couplings encountered here are weak and occur only in a 10–20 nm bandwidth. In a PBGF there are several resonators (in all-solid PBGFs these are, e.g., the high-index regions) and the mutual coupling between the same mode of these many resonators leads to the formation of the broader bands^30^.

## Chalcogenide M-type Fibers

**Figure 5:**
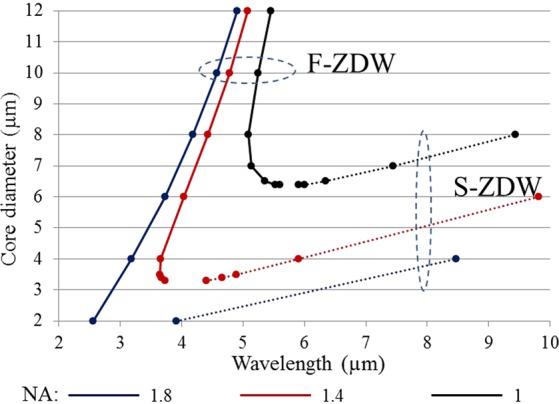
F-ZDW (solid curves) and S-ZDW (dotted curves) versus core diameter for the fundamental mode (LP_01_) of a conventional chalcogenide SIF for different NAs.

The investigations in the previous section have revealed the potential of M-type fibers for utilizing the LP_02_ mode for obtaining a core-guided mode with a shorter ZDW. In order to optimize and understand the advantage of M-type fibers over SIFs, it is important to do a comparative analysis of the first ZDWs (F-ZDWs) and second ZDWs (S-ZDWs) of M-type and SIFs with the same core diameters and NAs. Figure 5 shows a plot of the F-ZDW and S-ZDW of chalcogenide SIFs for different core diameter and NAs. The material dispersion given in Eqs (1–2) has been taken into account. In order to increase the NA, we again decreased the refractive index of the cladding by a factor independent of wavelength NA(λ)^2^ = n_1_(λ)^2^ − (n_2_(λ) − X)^2^ as mentioned in Section 2. It is important to understand that the NAs considered here are practically achievable and have been successfully fabricated^27,28,31,32^.

It is interesting to observe the trends in Fig. 5, with increasing core diameter F-ZDW and S-ZDW generally increase and a significant increase in the bandwidth (BW) with anomalous dispersion, defined as BW = (F-ZDW) − (S-ZDW), can be observed. Similarly, when increasing NA for a fixed core diameter, the F-ZDW decreases and the S-ZDW increases, which again leads to an increase in BW. In order to shift the F-ZDW to shorter wavelength, it is desirable to have a large NA and small core diameter thanks to the strong guidance of the light. However a combination of large NA and small core diameter leads to very small BW not suitable for broadband SC generation; moreover a small core diameter leads to reduced coupling efficiency and poor power handling. Based on Fig. 5 we can conclude that the F-ZDW remains higher than 5 μm and 3.6 μm for NAs of 1 and 1.4, respectively, irrespective of core diameter. In contrast, for an ultra-high NA of 1.8, the F-ZDW can be shifted below 3 µm for core diameters below 3.4 µm However, due to the generic variation of the ZDWs, the bandwidth is significantly degraded for core diameters below 3 µm, with the S-ZDW even being below the 4.5 µm loss edge of ZBLAN fibers, thereby taking away all the advantages of long wavelength guidance of chalcogenide fibers.

The above investigation verifies the limitation of using SIFs to achieve a dispersion profile suitable for broadband mid-IR SC generation using established ytterbium, erbium, and thulium pump laser technologies. Even the emerging Er:ZBLAN fiber laser technology at 2.8 µm seems to be useless as a pump source^33^. Therefore, it is desirable to improve the fiber design in order to at the same time have a larger core diameter and a shorter first ZDW, so that coupling efficiency and power handling can be increased.

**Figure 6:**
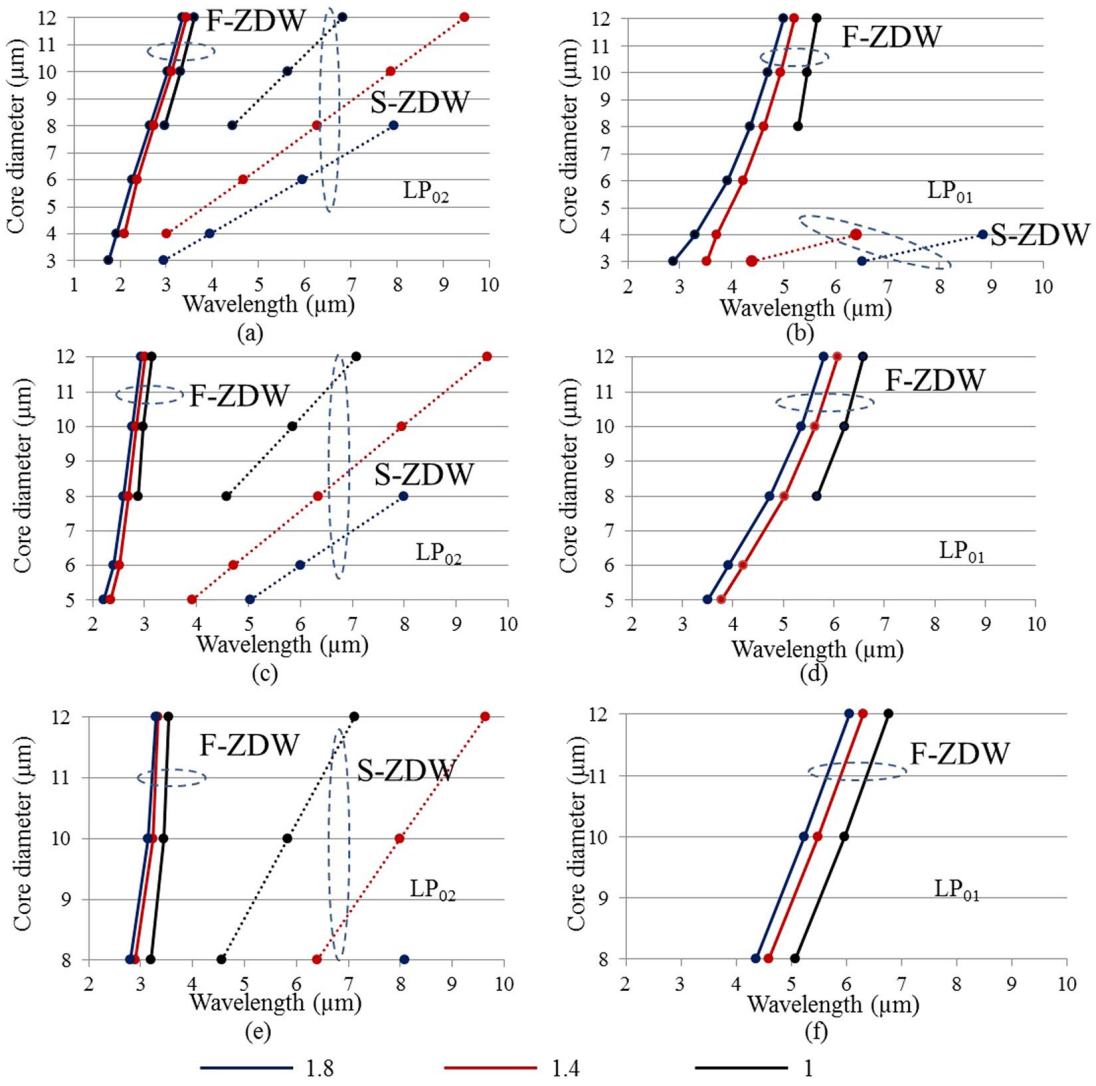
F-ZDW (solid curves) and S-ZDW (dashed curves) versus core diameter for the LP_02_ and LP_01_ mode of an M-type chalcogenide fiber for different NAs and ring thicknesses, while dn = 0.05 is fixed. M-type fiber demonstrated in (**a**–**d**), and (**e**,**f**) have t = 1 µm, t = 2 µm, and t = 3 µm, respectively.

**Figure 7:**
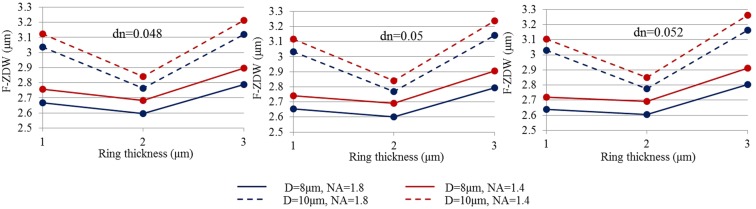
F-ZDW versus ring thickness for different core diameters (8 μm and 10 μm) and NAs (1.4 and 1.8) for varying dn (**a**) dn = 0.048, (**b**) dn = 0.05, and (**c**) dn = 0.052.

The goal is to shift the first ZDW to be below 2 µm in order to employ mature fiber laser technology or at least below 2.94 µm to use Er:ZBLAN fiber lasers^33^. However, any improvement in fiber design pushing down the ZDW of large core diameter chalcogenide fibers, will in fact be helpful. Figure 6 considers M-type fibers and shows the calculated F-ZDW and S-ZDW of the LP_01_ and LP_02_ modes for different D, t, and NA, but with dn fixed at 0.05. Figure 6(a–f) show the F-ZDW and S-ZDW of the LP_01_ and LP_02_ modes for t = 1 µm, 2 µm, and 3 μm respectively. A comparison of Figs 5, 6 shows the significantly shorter F-ZDW of the M-Type LP_02_ mode compared to the F-ZDW of the LP_01_ mode of a conventional SIF for all NAs and core diameters. The F-ZDW of the M-type fiber varies from 1.92 to 3.6 µm for all core diameters from 4 μm to 12 µm and NAs from 1 to 1.8. On the other hand the F-ZDW of a SIF varies from 3.2 µm to 5.5 µm for core diameters from 4 µm to 12 µm and NAs from 1 to 1.8. In addition to shifting the F-ZDW towards shorter wavelengths, another benefit arises as the larger core of the M-type fiber affords more pump power handling capacity. Figure 6 shows the dramatic improvement offered by the M-type fiber in shifting down the ZDW of a corresponding SIF with the same core diameter and NA by more than 1500 nm. Considering only core diameters above 4 µm for good power handling, we see that in fact even for the M-type fiber, no combinations of D ≥ 4 µm, NA ≤ 1.8, and t ≥ 1 µm allows a broad mid-IR SC by directly pumping with a 2 μm laser in the anomalous dispersion regime above the F-ZDW in a fiber with a S-ZDW larger than 6 μm. However, the M-type fibers do open the door for direct pumping by a 2.94 μm Er:ZBLAN fiber laser^33^. Figure 7 shows the F-ZDW versus the ring thickness t for different core diameters, NAs, and dn. We see that there seems to be an optimum value of t around = 2 µm at which the F-ZDW is a minimum and below 2.9 µm for all parameter combinations, which is suitable for using an Er:ZBLAN fiber pump laser. It is important that that for t = 2 µm even for a large core diameter of 10 µm and an NA of 1.4, which is achievable today^31^, the M-type fiber has an F-ZDW below 2.9 µm.

**Figure 8:**
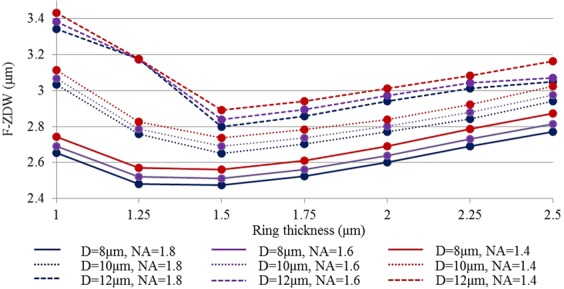
F-ZDW versus ring thickness for different core diameters (8 μm, 10 μm, and 12 μm) and NAs (1.4, 1.6, and 1.8) for fixed dn = 0.05.

In order to further zoom in on the optimum parameters with a minimum of F-ZDW and maximum S-ZDW, we performed more simulations. Figure 8 shows the F-ZDW for different ring thicknesses between 1 and 2.5 μm, core diameters D = 8, 10, and 12 μm, and NA = 1.4, 1.6, and 1.8. In Fig. 8, we did not investigate the impact of the dn variation, as Fig. 7 already showed the negligible impact of a change in dn from 0.048 to 0.052 had on the F-ZDW. Figure 8 shows that the minimum is closer to t = 1.5 µm and that the F-ZDW remains lower than 3 μm for most of the fiber parameters, thereby ensuring a possibility to be pumped by a 2.94 μm laser. In terms of the S-ZDW, it remained longer than 7.92 µm for t ≥ 1.5 µm and all parameters other than D = 8 μm and NA = 1.4 and 1.6. In order to achieve a broadband spectrum, it is good to look for parameters fulfilling the following conditions: F-ZDW ≤ 3 and S-ZDW ≥ 7.9 μm. The following sets of parameters out of the parameters shown in Fig. 8 fulfil these conditions, (1) D = 8 μm, NA = 1.8, irrespective of t, (2) D = 10 μm, irrespective of t and NA other than t = 1 μm-1.25 μm and NA = 1.4, and (3) D = 12 μm, t = 1.5, 1.75, and 2 μm, and irrespective of NA.

The optimum M-type fiber design considered here with an F-ZDW of 2.6 μm can in fact be pumped with a 2 μm broadband laser by using in-amplifier supercontinuum generation^34,35^. Recently, high power SC sources extending up to ~2.7 μm in all-fiber thulium doped fiber amplifier (TDFA) have been reported^36^. Further, SC sources extending up to 3.2 μm in a Germania doped silica fiber have also been achieved by pumping with a 1550 nm laser source in an all-fiber configuration^37,38^. In particular the latter could be promising to pump most of the M-Type fibers shown in Fig. 8. In recent years, a cascaded pumping scheme has also emerged as a versatile tool to compensate for the unavailability of fiber lasers above 2 µm^36,39,40^. A typical example of cascaded pumping of chalcogenide fibers in mid-IR SC generation is pumping first a ZBLAN fiber using a conventional 2 µm laser and then couple the supercontinuum output of the ZBLAN fiber (normally extending up to ~4.2 µm) into a chalcogenide fiber for further broadening. It is quite obvious that a SIF may not be an exciting option in this cascaded configuration due to its stringent trade-off between core diameter, short F-ZDW, and large BW as shown in Fig. 5. In a recent experimental study of such cascaded configurations, a suspended core chalcogenide fiber of ~4.5 µm diameter having a ZDW of ~3.5 µm was used, which has limited coupling efficiency due to the small core and very bad power handling capability because the 3 large holes around the core thermally isolates the core^40^. It is very important to make a note here that, there are several other combinations of t and dn having ZDW lower than 3.5 μm being suitable for cascaded configuration. An improved M-type fiber design having a shorter ZDW with a larger core diameter than a SIF will open the door to power scaling in this cascaded scheme, as more power from the ZBLAN fiber output will be in the anomalous dispersion regime of the chalcogenide fiber.

**Figure 9:**
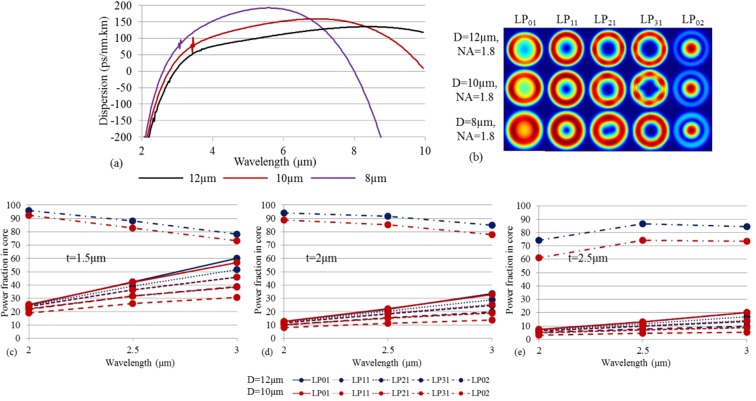
(**a**) Dispersion versus wavelength for varying core diameter. The other parameters are t = 2 μm, NA = 1.8, and dn = 0.05. (**b**) The electric field intensity profile of the several modes for different core diameter at 3 µm wavelength. (**c**–**e**) show the power fraction in core for different modes at different wavelengths for varying t. The other parameters are NA = 1.8 and dn = 0.05.

Going deeper into the properties of the M-Type fiber it is extremely important to note that, in order to excite the LP_02_ mode without exciting the LP_01_ and other modes, it is necessary to choose the thickness (t) and the refractive index (dn) of the ring, so that all these unwanted modes lie in the high-index ring with much less power in the core than the LP_02_ mode. In Fig. 9 we consider M-type fibers with NA = 1.8, t = 2 μm, and dn = 0.05, and varying core diameters From the dispersion profiles we again see the narrow-band small spikes in the 2 to 4 µm region, originating from the coupling between LP_31_ and LP_41_ modes with the LP_02_ mode. Figure 9(b) shows the profiles of the electric field intensity of various modes for different core diameters at λ = 3 μm. In particular we see that indeed the LP_02_ mode is core-guided, but we also see that in fact the LP_01_ mode, while still having most power in the ring, is starting to have significant power in the core as the core diameter is decreased from 12 µm to 8 µm. In Fig. 9(c–e) we study this point by plotting the fraction of power in the core of the LP_02_ mode and the main ring-based modes (LP_01_, LP_11_, LP_21_, LP_31_) for a ring thickness of t = 1.5 μm, 2 μm, and 2.5 μm, and core diameters of D = 10 μm and 12 μm. The other parameters are NA = 1.8 and dn = 0.05. Figure 9(c–e) demonstrates that when the core diameter and ring thickness are sufficiently large then the power of the ring-guided modes in the core is so small that the LP_02_ mode effectively is the only core-guided mode. For the small thickness of t = 1.5 µm, however, the power in the core of the ring-guided modes start to become comparable to that of the LP_02_ mode at longer wavelength, which means that significant coupling can be anticipated. Combined with the ZDW investigations it appears that a thickness of 2 µm and a core diameter of 10 µm is reasonable for 2.9 µm pumped broadband mid-IR SC generation.

**Figure 10:**
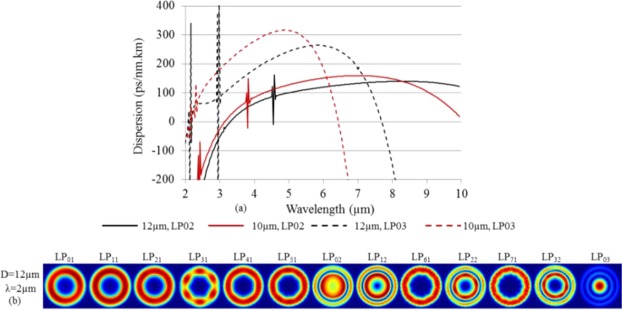
(**a**) Dispersion of the core-guided modes LP_02_ and LP_03_ modes versus wavelength for two different core diameter. The other parameters are t = 3 μm, NA = 1.8, and dn = 0.05. (**b**) The electric field intensity profile of several modes for D = 12 μm, t = 3 μm, NA = 1.8, and dn = 0.05.

In view of the beneficial properties of the core-guided LP_02_ mode in the M-type fiber an interesting question could be whether the also core-guided LP_03_ mode could be used to achieve an even shorter F-ZDW. In order to explore this, we further extended the study to a thickness of t = 3 µm. Figure 10(a) shows the calculated dispersion for the core-guided LP_02_ and LP_03_ modes for two core diameters D = 10 μm and 12 μm. The other parameters are: NA = 1.8 and dn = 0.05. The F-ZDW for the LP_03_ mode is between 2.1 and 2.2 μm. It is difficult to determine the exact value due to a very strong spike due to coupling with ring modes in this range, much stronger than what has been seen for the LP_02_ mode. Although the LP_03_ mode has much shorter F-ZDW than LP_02_ (>3 μm) but it comes at the cost of a shorter S-ZDW, which is lower than 7.7 μm for D = 12 μm. Figure 10(b) shows the profile of the electric field intensity of different modes for D = 12 μm and demonstrates that the LP_02_ and LP_03_ modes are core-guided at λ = 2 μm, while the other modes are ring-guided. At a first look exploiting the LP_03_ mode could seem a tempting option for mid-IR SC generation, as it potentially allows using a 2 μm pump laser source. However, the results of Fig. 10 show that the LP_03_ mode suffers more from resonant couplings with ring-guided modes and from a short S-ZDW, than the LP_02_ mode. A detailed investigation would therefore be required to judge the advantages of the LP_03_ mode over the LP_02_ mode. Furthermore, for M-type silica fibers, researchers have used two high index layer in place of one to exploit the LP_03_ modes^10^, however it might be fairly difficult to have two high index layers in case of chalcogenide compared to the silica glass based fibers. Therefore, exploitation of LP_03_ using two index layers has been left here for future work.

## ZBLAN M-type Fibers

**Figure 11:**
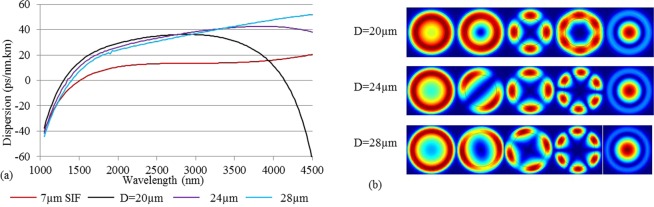
(**a**) Dispersion profile of the LP_01_ mode of a ZBLAN SIF with D = 7 µm and NA = 0.3, and the LP_02_ mode of an M-type ZBLAN fiber with D = 20 µm, 24 µm, and 28 µm, t = 4 µm, dn = 0.005, and NA = 0.3. (**b**) The electric field intensity profile of several modes for all three M-type fibers at 1.55 µm wavelength.

In Fig. 11 we extend the study of M-type fibers to ZBLAN fibers. Here we have assumed a core having the composition 53%ZrF_4_–20%BaF_2_–4%LaF_3_–3%AlF_3_–20%NaF, whose refractive index was obtained using the Sellmeier equation reported in^41^. We obtained the index of the cladding as n_cl_(λ)^2^ = n_c_(λ)^2^ − NA and of the high-index ring as n_r_(λ) = n_c_(λ) + dn. Figure 11(a) shows the dispersion profile of the LP_01_ mode of a ZBLAN based SIF with D = 7 µm and NA = 0.3, and of the LP_02_ mode of three M-type ZBLAN fibers with D = 20 µm to 28 µm, t = 4 µm, dn = 0.005, and NA = 0.3. Figure 11(b) shows the profile of the electric field intensity at 1.55 μm for the M-type fibers with different core diameters (D = 20 µm, D = 24 µm, and D = 28 µm). The mode profiles show that all modes lower than the LP_02_ are confined to the ring, which ensures the excitation of the LP_02_ mode as the fundamental mode is possible. One can further see how the ring-guided modes become increasingly confined to the ring as the core diameter is increased.

Figure 11(a) shows that the ZDW increases with increasing core diameter. It is interesting to note that, the ZDW for none of these fibers exceeds 1.4 µm; on the contrary a 7 µm core diameter SIF with 0.3 NA has a ZDW of 1.5 µm. It is important to keep in mind that, with increasing core diameter, the ZDW will shift to a longer wavelength; hence it can be concluded that a 7 µm core diameter (for NA = 0.3) is towards the maximum core diameter of a SIF that can be pumped efficiently by a 1.55 µm source in the most efficient anomalous dispersion regime. On the contrary, an M-type fiber (with same NA = 0.3) has a ZDW lower than 1.5 µm, even with a core diameter of 28 µm (four times larger than the 7 µm core SIF.) This again shows the clear opportunity of pumping the M-type ZBLAN fiber with a 1.55 µm source and using the much larger core to handle more power^42^.

## Conclusion

Our results have demonstrated that M-type fibers have tremendous potential for mid-IR supercontinuum generation applications through the capability of having HOMs, such as the LP_02_ or LP_03_ modes as a fundamental mode and consequently having shorter ZDWs with larger core diameters. In case of chalcogenide fibers, the ZDWs can be shifted down into the range of 2 µm to 3 µm wavelength. The above investigations show the great potential of the M-type fiber to be pumped by a 2.8 μm Er:ZBLAN laser, a broadband Tm and Ho doped fiber laser, a highly non-linear Germania doped fiber having broadband supercontinuum spectrum output, and a supercontinuum output of a ZBLAN fiber. Here, due to the shorter ZDW of the M-type fiber compared to its counterparts such as SIFs or suspended core fibers, more solitons from a supercontinuum pump source (such as a supercontinuum output from ZBLAN fiber) can be pushed into anomalous dispersion leading to more output power towards longer wavelengths. Further investigations revealed the tremendous potential of using a 1.55 µm laser source as a direct pump source for ZBLAN based fiber with a dramatic increase in core diameter. The M-type fiber can prove a milestone in the development of all-fiberized high power supercontinuum sources pumped by a low cost 1.55 µm pump source. Furthermore, M-type fibers will offer the advantages of easy fabrication and handling compared to air-hole based photonic crystal fibers.

## Methods

In this study, we use finite element method based commercially available software known as COMSOL MULITPHYSICS for numerical simulations. The effective refractive index of different modes was calculated with respect to wavelength with a step size of 1 nm. A fine mesh of triangular elements with a maximum size of 1 µm was used for entire calculations. The smaller is the mesh size, lower is the error of calculations, however it comes at the cost of computation time. For our calculations, the chosen mesh size is lower than any wavelength operated: mesh size (max) = 1 µm < any operating wavelength between 2 to 10 µm to maintain high standards of calculations. The dispersion, the second order derivative of effective refractive index with respect to wavelength (1), was calculated using finite difference method with a fourth-order accuracy using following expression (2).

$${D}_{\lambda }=\frac{-\lambda }{c}\ast \frac{{d}^{2}n}{d{\lambda }^{2}}$$

$${D}_{\lambda (0)}=\frac{-{\lambda }_{0}}{c}\ast \frac{-2{n}_{-2}+32({n}_{-1})-60({n}_{0})+32({n}_{1})-2({n}_{2})}{24\Delta {\lambda }^{2}}$$

λ_0_ = Operating wavelength, ∆λ = step-size of wavelength, n_−2,_ n_−1_, n_0_, n_1_, and n_2_ are the effective indices at λ − 2∆λ, λ − ∆λ, λ, λ + ∆λ, and λ + 2∆λ operating wavelength respectively, and c is the speed of light in vacuum.

The unit of Dispersion (D_λ_) is ps/nm.km, therefore λ is on nm scale and c, speed of light is on km/ps scale.
